# Combined genetic mutations and DNA-methylated genes as biomarkers for endometrial cancer detection from cervical scrapings

**DOI:** 10.1186/s13148-019-0765-3

**Published:** 2019-11-28

**Authors:** Phui-Ly Liew, Rui-Lan Huang, Tzu-I Wu, Chi-Chun Liao, Chien-Wen Chen, Po-Hsuan Su, Hui-Chen Wang, Yu-Chun Weng, Hung-Cheng Lai

**Affiliations:** 1 Department of Pathology, Shuang Ho Hospital, Taipei Medical University, New Taipei, Taiwan; 2 Department of Pathology, School of Medicine, College of Medicine, Taipei Medical University, Taipei, Taiwan; 3 Department of Obstetrics and Gynecology, Shuang Ho Hospital, Taipei Medical University, New Taipei, Taiwan; 4 Translational Epigenetic Center, Shuang Ho Hospital, Taipei Medical University, New Taipei, Taiwan; 5 Department of Obstetrics and Gynecology, School of Medicine, College of Medicine, Taipei Medical University, Taipei, Taiwan; 6 Department of Obstetrics and Gynecology, Wan Fang Hospital, Taipei Medical University, Taipei, Taiwan; 7 Department of Clinical Pharmacology, Xiangya Hospital, Central South University, Changsha, 410008 People’s Republic of China; 8 Institute of Clinical Pharmacology, Central South University; Hunan Key Laboratory of Pharmacogenetics, Changsha, 410078 People’s Republic of China; 9 New Taipei, Taiwan

**Keywords:** Endometrial cancer detection, Cervical scrapings, Mutation, Methylation, Biomarkers

## Abstract

**Background:**

Endometrial cancer is a common gynecologic cancer. Noninvasive molecular biomarkers for triage of high-risk patients for invasive procedures are needed. Based on the success of cytological Pap smear screening, cervical scrapings are a good source of DNA for molecular testing. In addition to genetic lesions, DNA methylation is a promising biomarker. We assessed the usefulness of combining genetic and epigenetic biomarkers from cervical scrapings to detect endometrial carcinomas.

**Methods:**

We performed a retrospective case–control study of 96 consecutive cervical scrapings from patients with abnormal uterine bleeding who underwent surgery for diagnostic evaluation. Thirty and 16 cases were diagnosed with type I and type II endometrial cancers, respectively. The remaining non-cancer cases included normal endometrium (*n* = 12), benign uterine lesions (*n* = 20), and endometrial hyperplasia (n = 18). Quantitative methylation-specific PCR and mass spectrometry were used for DNA methylation and genetic mutation analysis. Logistic regression was used to evaluate the clinical performance of these candidate biomarkers.

**Results:**

We tested the effectiveness of the methylation status of four genes (*BHLHE22*, *CDO1*, *TBX5*, and *HAND2*) in endometrial cancer detection. The area under the receiver operating characteristic curves ranged from 0.703 to 0.878, and panels of hypermethylated *BHLHE22*/*CDO1*/*HAND2* (87.0% sensitivity and 86.0% specificity) and *BHLHE22*/*CDO1*/*TBX5* (89.1% sensitivity and 80.0% specificity) showed significant differences and could distinguish benign from malignant endometrial lesions. The sensitivity and specificity in endometrial cancer detection for *BHLHE22*/*CDO1* were 84.8% and 88.0%, respectively. Both type I and II endometrial carcinomas could be detected using a *BHLHE22*/*CDO1*-based methylation profile, suggesting that they may have common epigenomes. Moreover, *PTEN* and *TP53* mutations were found in 63.3% of type I and 93.6% of type II endometrial cancers. Unexpectedly, *PTEN* and *TP53* mutations were commonly found in cervical scrapings of the normal endometrium (25% and 33.3%, respectively) and in cases with benign uterine lesions (10% and 50%, respectively). Finally, combinations of any one mutation of *PTEN* and *TP53* mutations had a sensitivity of 91.3%, but a specificity of only 42.0%.

**Conclusions:**

Adding *PTEN*/*TP53* mutation testing to *BHLHE22*/*CDO1*-based methylation testing did not improve the detection of endometrial cancer.

## Background

Endometrial cancer (EC) is the most common female genital tract malignancy in developed countries [1]. Although abnormal or dysfunctional uterine bleeding is the most frequent symptom of EC, only 10% of postmenopausal women with this symptom have EC. The most common diagnostic test for EC is transvaginal ultrasound, which measures the thickness of the endometrium. Unfortunately, transvaginal ultrasound cannot reliably distinguish between benign and malignant lesions [2]. Moreover, a cutoff value for an endometrial thickness that warrants further hysteroscopy remains under debate. Thus, invasive procedures to obtain endometrial tissues by fractional dilatation, curettage, and hysteroscopic biopsy remain necessary. However, the chance of diagnosing EC using current methods is low, even in symptomatic patients. Consequently, noninvasive molecular markers with acceptable accuracy for EC screening, at least in symptomatic women, are much needed.

The endometrium is a highly proliferative and cyclically regenerative tissue, which makes it vulnerable to genetic and epigenetic changes. While estrogen drives endometrial cell proliferation, progesterone inhibits it and causes cell differentiation. Conditions associated with a functional dominance of estrogen over progesterone increase the risk for both endometrial hyperplasia and EC. EC is broadly classified into two histotypes: type I, which consists of predominantly endometrioid adenocarcinomas, and type II, which incorporates serous-type, clear-cell, and poorly differentiated carcinomas. The proposed hyperplasia-to-carcinoma sequence for type I EC involves unopposed estrogen activity with subsequent mutations or alterations in pro-growth molecular pathways. Moreover, genomic data from The Cancer Genome Atlas defined four molecular subtypes of EC [3]. The four molecularly defined cancer subtypes are: DNA polymerase ε exonuclease domain mutation, microsatellite-instable, microsatellite-stable with fewer copy-number alterations (CNAs), and microsatellite-stable (serous-like) with more CNAs. A categorization of ECs into one of these four subgroups could potentially provide individuals with prognostic and predictive information [4].

Unfortunately, the application of such genetic information in endometrial screening remains limited. Loss of function of the phosphatase and tensin homologue (*PTEN*) tumor suppressor heralds the beginning of multistep carcinogenesis, and its somatic mutation and/or deletion is the most common genetic change in endometrial endometrioid adenocarcinoma [5]. *PTEN* lesions are present in 83% of sporadic EC cases [6] and have been proposed to serve as diagnostic markers for endometrial precancerous lesions [7]. In addition to *PTEN*, approximately 25% of all ECs have been found to harbor mutations in the tumor suppressor *TP53* [8]. Although both *PTEN* and *TP53* drive carcinogenesis in many malignancies, the mechanistic role of both genes in endometrial carcinogenesis has not been fully elucidated [9].

A recent investigation revealed the feasibility of testing such genetic mutations in cervical scrapings (“Pap smears”) for EC detection [10, 11]. In addition to genetic anomalies, epigenetic alternations are also involved in complex cancer development [12]. Aberrant DNA methylation-associated transcriptional silencing in tumor suppressor genes is commonly observed in human cancers [13]. Two predominantly global methylation patterns in cancer have been generally acknowledged: DNA hypermethylation of specific gene promoters, leading to gene silencing (“localized hypermethylation”), and loss of methylation within highly repeated DNA sequences, leading to unstable genomes and aberrant expression of oncogenes (“global hypomethylation”). Thus, understanding epigenetic regulation in EC progression may open new avenues for EC detection.

In light of the above, epigenetic lesions could potentially serve as early detection biomarkers for EC. For example, an epigenome-wide methylation analysis revealed that Heart and Neural Crest Derivatives Expressed 2 (*HAND2*) was one of the most commonly hypermethylated and silenced genes in EC [14]. Li and colleagues also demonstrated that *HAND2* plays a major role in endometrial stromal-epithelial signaling [15], and that, in the presence of progesterone, endometrial epithelial cell proliferation inhibits *HAND2* upregulation in endometrial stroma. Thus, *HAND2* methylation is a common and crucial molecular alteration in EC that could hold clinical implications [16, 17]. The feasibility of *HAND2* methylation testing in cervical scrapings and its value for EC detection remain undetermined. Our previous comprehensive methylomics approach identified in EC tissues a panel of highly methylated genes, including *BHLHE22/CDO1/CELF4*, that were detectable in cervical scrapings [18]. Such studies provide proof-of-concept for new means of EC screening using epigenetics analyses.

The combination of genetic and epigenetic markers from cervical scrapings for EC detection is logical and appealing. However, to our knowledge, no studies have tested such an approach. Therefore, the aim of the present study was to test whether the use of panels of combined epigenetic and genetic markers derived from cervical scrapings could improve EC detection.

## Methods

### Clinical samples

Female participants (age range, 30–80 years) were enrolled in our case–control retrospective study from November 2015 to September 2017. These women had cervical scrapings performed because of abnormal or dysfunctional uterine bleeding, followed by surgery at the Taipei Medical University-Shuang Ho Hospital, New Taipei City. This study was approved by the Institutional Review Board of the Taipei Medical University-Shuang Ho Hospital, in accordance with the Declaration of Helsinki 2000 (Protocol no. N201902024). Informed consent was obtained from all participants. Age, histologic type/grade of lesion or tumor, and International Federation of Gynecology and Obstetrics stage were extracted from the hospital records. Following collection, cervical scrapings were immediately placed in a RNA*later* Stabilization Solution (Ambion, Thermo Fisher Scientific) and stored at − 80 °C for future analysis.

### Methylation analysis of *BHLHE22*, *CDO1*, *HAND2*, and *TBX5* genes

Genomic DNA was extracted from the cervical scrapings using the QIAmp DNA Mini Kit (QIAGEN, Hilden, Germany), 800 ng of which was modified with bisulfite using an EZ DNA Methylation Kit (D5008; Zymo Research, Irvine, CA, USA) according to the manufacturer’s instructions, and then dissolved in 70 μL nuclease-free water. PCR products and quantitative methylation-specific polymerase chain reaction (qMS-PCR) were performed using a LightCycler 480 SYBR Green I Master (Roche, Penzberg, Germany). Reactions were carried out in 20 μL containing 2 μL bisulfite-converted DNA, 250 nmol/L of each primer, and 10 μL Master Mix using the following thermal profiles: 95 °C for 5 min (initiation), 50 cycles of 95 °C for 10 s, 60 °C for 30 s, and 72 °C for 30 s (amplification), and a final extension step at 72 °C for 5 min. All gene amplifications were conducted using duplicate specimens. To calculate a relative target amount, only the respective crossing point (Cp) values of the target, the reference gene for each sample, and a calibrator need to be determined using LightCycler software. To normalize the input DNA in each methylation-independent assay, we used the amount of a non-CpG region of a type II collagen gene (*COL2A1*) as internal reference [19]. DNA methylation levels were estimated by the difference in crossing point (ΔCp) values using the following formula: Cp of target − Cp value of *COL2A1*. We considered and simplified the percentage of methylated reference for each result using the ΔCp values for detecting DNA methylation [20, 21]. The analytical sensitivities of candidate genes are shown in Additional file 1: Figure S1. Linear regression analysis was used to assess assay linearity. The serial dilution of *COL2A1* showed that the DNA temple was 118 copies; the mean Cp value of *COL2A1* was 35.8. Therefore, we defined Cp values of *COL2A1* > 36 as not detected for each candidate gene in our samples. *BHLHE22-*, *CDO1-*, and *TBX5*-specific primers were designed using Oligo 7.0 Primer Analysis software (Molecular Biology Insights, Inc.). We used the primer sequences of *HAND2* described in a previous study [14].

### Somatic mutation detection and analysis of *TP53* and *PTEN* genes

To identify *PTEN* and *TP53* exomic mutations, we sequenced all exons of those two genes using the Illumina HiSeq2500 high-throughput genome sequencer (Illumina, Inc., San Diego, CA, USA). Next, 40 ng of DNA from each individual was used to construct a DNA library of 43 target regions using the QIAseq targeted DNA system (QIAGEN). Briefly, DNA was enzymatically fragmented and end-repaired in a 25 μL solution containing 2.5 μL 10× fragmentation buffer and 5 μL fragmentation enzyme mix. The reaction was carried out at 4 °C for 1 min, 32 °C for 24 min, and 65 °C for 30 min. Immediately after the reaction, 10 μL 5× ligation buffer, 5 μL DNA ligase, 2.8 μL of 25 μM bar-coded adapters, and water were added for a total volume of 50 μL. The reaction was then continued at 20 °C for 15 min. To ensure the complete removal of free barcoded adapters, each reaction was purified twice using a bead-system (QIAGEN). In a total volume of 20 μL, purified DNA was mixed with 10 nM of each target primer, 400 nM forward primer, 1× PCR buffer, and 0.8 μL HotStarTaq DNA polymerase. The PCR enrichment conditions were: 95 °C for 13 min, 98 °C for 2 min, six cycles of 98 °C for 15 s, 65 °C for 15 min, and 72 °C for 5 min. Each reaction mixture was purified to remove unused primers. The enriched DNA was combined with 400 nM universal primer, 400 nM index primer, 1× PCR buffer, and 1 μL HotStarTaq DNA polymerase in a total volume of 20 μL. The universal PCR conditions were: 95 °C for 13 min, 98 °C for 2 min; 20 cycles of 98 °C for 15 s, 60 °C for 2 min, and 72 °C for 5 min. The DNA library was then purified and pooled for sequencing (2 × 100 base pairs [BPs]). The raw output from each individual scraping was > 100 Mb, with an average target region depth > 30,000×. The sequence of each read was trimmed based on its quality score (Q30), and a length < 45 BPs of each read was discarded from the following analyses. The reads were aligned to the human hg19 reference genome using BWA-MEM (http://bio-bwa.sourceforge.net/), and variants were called using GATK Unified Genotyper (GATKLite version 2.3–9) [22]. After variant calling, Variant Effect Predictor (http://grch37.ensembl.org/Homo_sapiens/Tools/VEP) was used to annotate the identified variants. We then selected the confidence of the mutations using alternate allele frequencies ≥ 0.3 as a cutoff and removed synonymous substitutions (i.e., silent mutations in the encoded protein). All *PTEN* and *TP53* mutations were displayed using MutationMapper software (https://www.cbioportal.org/mutation_mapper), as shown in Fig. 2a [23, 24]. The presence of a major mutation in each individual gene is shown as the maximum value of alternate allele frequencies in Fig. 2b.

### Statistical analysis

Based on previous work, the area under the receiver operating characteristic curve (AUC-ROC) of hypermethylated *HAND2* in the normal endometrium vs. EC tissues was 0.9 [14]. Consequently, we assumed that the AUC-ROC of hypermethylated *HAND2* in cervical scrapings from normal and type I EC patients was 0.8. The null hypothesis AUC-ROC, type I error (α), and type II error (β) values were 0.5, 0.01, and 0.05, respectively, and the ratio of sample sizes of normal vs. type I EC tissue was 1.0, requiring at least 28 cases in both groups. We then added three samples to both groups to avoid a 10% failure rate in subsequent tests. Additionally, we simultaneously examined gene mutations and DNA methylation levels in DNA isolated from cervical scrapings from patients with endometrial hyperplasia (*n* = 18) and type II EC (*n* = 16). The Mann–Whitney nonparametric *U* test and the Kruskal–Wallis test were used to identify significant differences in methylation levels between two categories and more than two categories, respectively. The associations between categorical clinical variables and methylation levels/genetic mutations were identified by the chi-square test for 2-by-2 categories. The combinations of methylated DNA levels were calculated using a logistic regression model, and each gene was weighted by a coefficient value. The cutoff values were evaluated by AUC-ROC analysis with the Youden method. A two-tailed *P* value ≤ 0.05 was considered significant.

## Results

### Hypermethylation of *BHLHE22*, *CDO1*, *HAND2*, and *TBX5* can be detected in cervical scrapings of patients with ECs

**Fig. 1:**
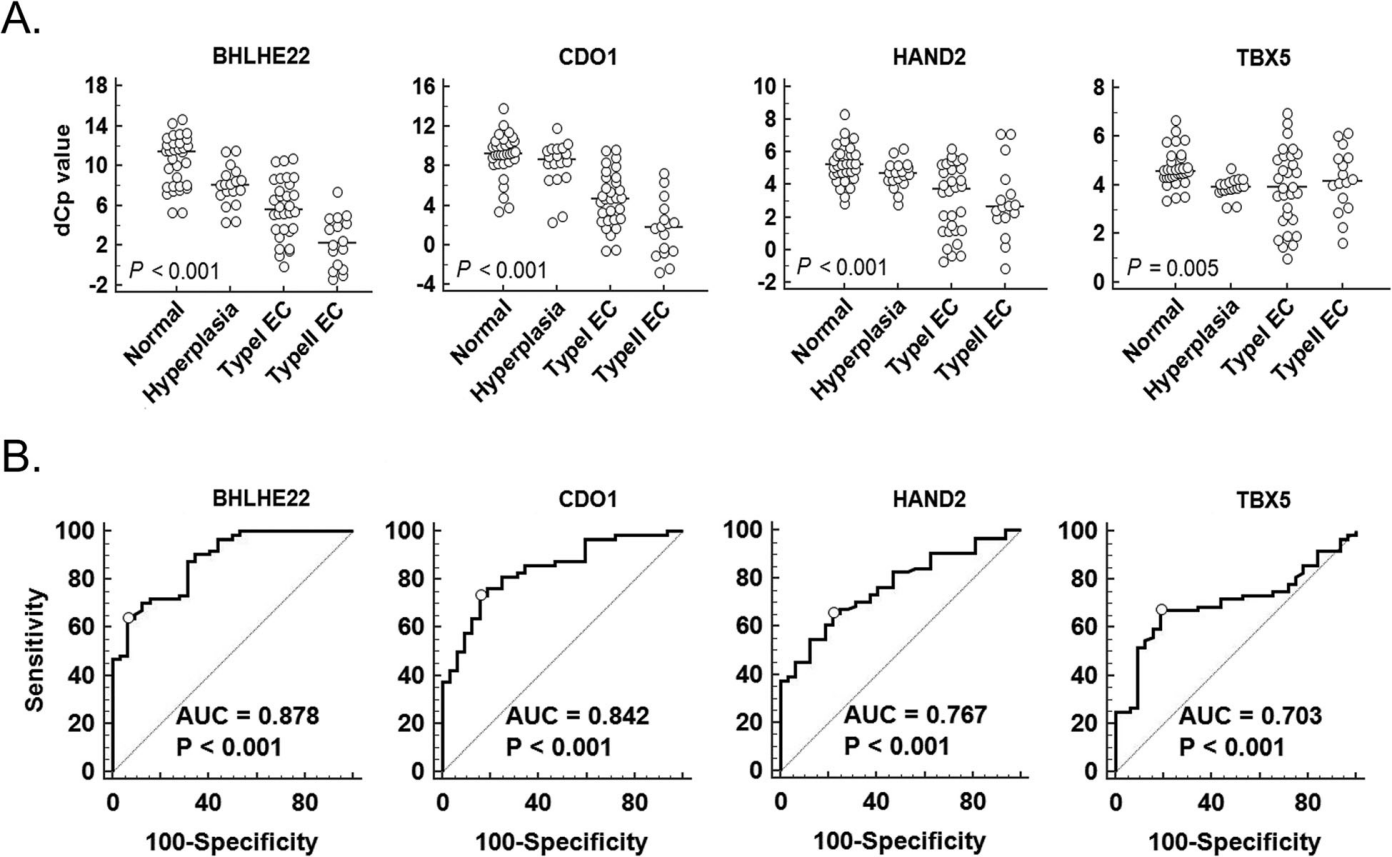
DNA methylation levels for four candidate genes detected by quantitative methylation-specific polymerase chain reaction (qMS-PCR) in 96 cervical scrapings. **a** DNA methylation levels are displayed as the difference in crossing point (ΔCp) values for each candidate gene. Dot plots indicate the distribution of ΔCp values for *BHLHE22*, *CDO1*, *HAND2*, and *TBX5*. Horizontal bars in the middle of the scattered dots indicate the average methylation levels. The lower the Cp values, the higher the gene methylation status. *P* values were calculated using the Kruskal–Wallis test. **b** Area under the receiver operating characteristic curve (AUC-ROC) for the DNA methylation status of the four candidate genes in cervical scrapings. *P* values were < 0.001 for all analyses, and ≤ 0.5 for the comparison of AUC-ROCs

Table 1 shows the clinical and pathological features of 96 patients from whom cervical scrapings and subsequent uterine tissue specimens were collected. These included scraped samples of normal endometrium (*n* = 12), benign diseases (*n* = 20), endometrial hyperplasia (*n* = 18), type I EC (*n* = 30), and type II EC (*n* = 16). The methylation levels of those four genes, in terms of ΔCp values, are displayed as dot plots in Fig. 1a. The lower the ΔCp value, the higher the gene’s methylation status. *BHLHE22*, *CDO1*, and *HAND2* methylation increased significantly from normal endometrium to endometrial hyperplasia, type I ECs, and type II ECs (*P* < 0.001) (Fig. 1a). Next, we calculated the AUC-ROCs for these four genes to determine the sensitivity and specificity of methylated DNA for cancer detection in cervical scrapings. The results ranged from 0.703 to 0.878 (Fig. 1b). *BHLHE22* performed the best of all tested genes.

**Table 1:** Demographics of clinical samples

Variables	Normal endometrium	Benign diseases	Hyperplasia	Endometrial cancer (EC)	
				Type I	Type II
Number of cases	12	20	18	30	16
Age (years)	53.4 ± 5.6	46.2 ± 5.8	46.4 ± 6.5	55.3 ± 6.9	59.6 ± 8.7
Subtypes					
Adenomyosis		1 (5%)			
Leiomyoma		10 (50%)			
Adenomyosis and leiomyoma		9 (45%)			
Endometrial hyperplasia			12 (42.9%)		
Atypical endometrial hyperplasia			6 (33.3%)		
Histotypes of cancer					
Endometrioid				30 (100%)	3 (18.8%)
Serous				0	6 (37.5%)
Others				0	7 (43.8%)
FIGO stage of cancer					
I				28 (93.3%)	10 (62.5%)
II				0	1 (6.2%)
III				1 (3.3%)	3 (18.8%)
IV				1 (3.3%)	2 (12.5%)
Histological grade of cancer					
G1				20 (66.7%)	4 (25.0%)
G2				8 (26.7%)	2 (12.5%)
G3				0	9 (56.2%)
Unknown				2 (6.7%)	1 (6.2%)

### *PTEN* and *TP53* mutations are commonly detected in cervical scrapings from patients with a wide range of endometrial lesions

**Fig. 2:**
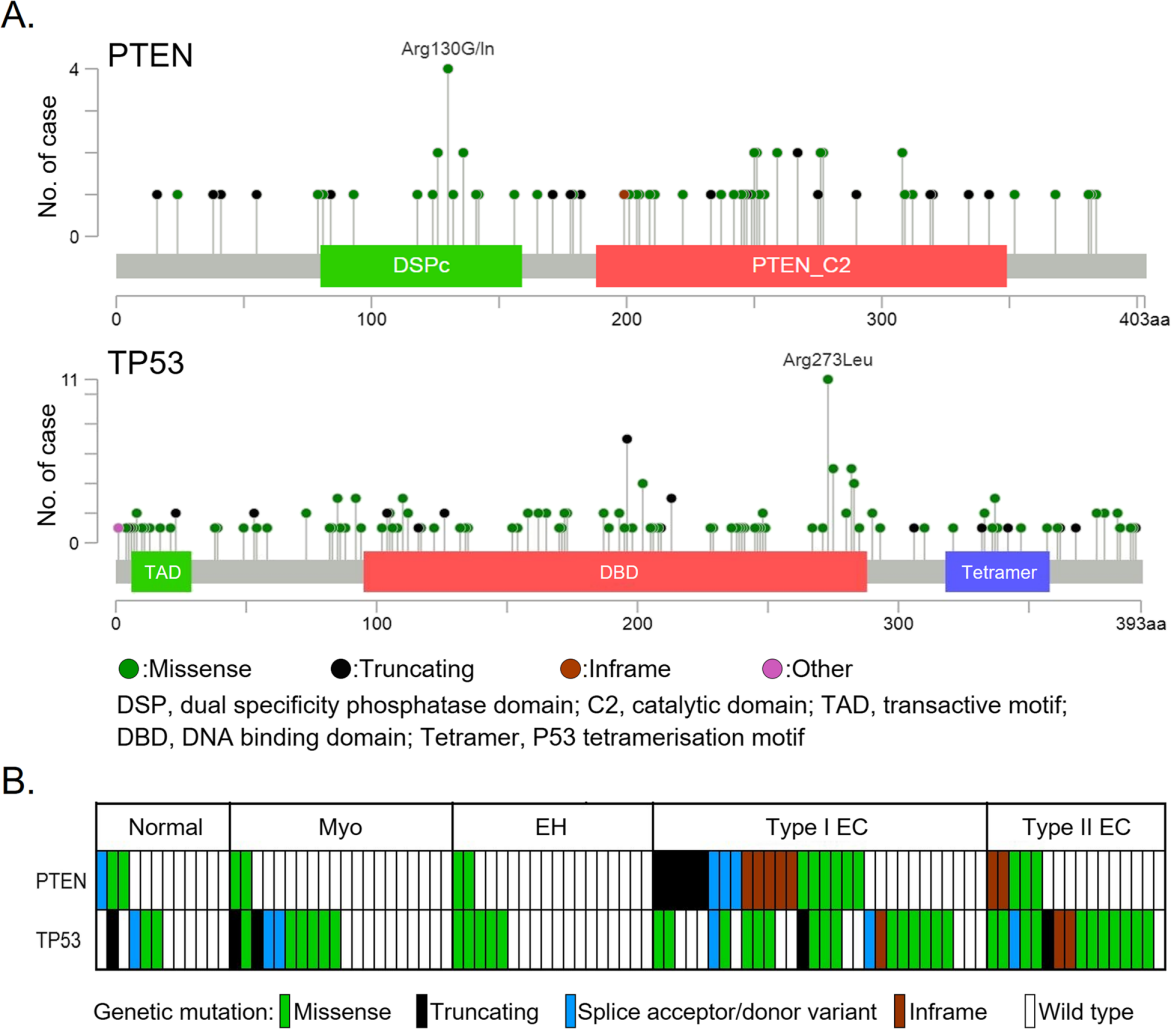
Localization of mutations in *PTEN* and *TP53* gene sequences in the patient cohort. **a** The distribution and spectrum of *PTEN* (top) and *TP53* (bottom) mutations are shown. The presence of a mutation is shown on the *x*-axis (lollipop), and the number of cases and their frequency of mutations are shown on the *y*-axis. Missense mutations are presented in green, truncating (“nonsense”) mutations in black, and in-frame mutations in brown. **b** The prevalence of *PTEN* and *TP53* mutations in normal endometrium (normal), leiomyoma (Myo), endometrial hyperplasia (EH), atypical endometrial hyperplasia (AEH), type I endometrial cancer (EC), and type II EC are shown

Targeted sequencing of *PTEN* and *TP53* revealed mutations across the whole exome of each gene in the control group (normal endometrium) and in the disease groups without hot spots (Fig. 2a). As expected, *PTEN* mutations were common in 63.3% of type I ECs, and *TP53* mutations in 93.6% of type II ECs (Fig. 2b). *TP53* mutations were also common in type I ECs (63.3%), and these were primarily missense mutations. Unexpectedly, *PTEN* and *TP53* mutations were commonly found in cervical scrapings of control patients and those with benign uterine lesions. *PTEN* and *TP53* mutations could be detected in controls (25% and 33.3%, respectively) and patients with benign uterine lesions (10% and 50%, respectively) (Fig. 2b). Simultaneous *PTEN* and *TP53* mutations correlated with disease progression from 8.3% (controls), to 10% (benign uterine lesions), to 34.7%, in EC cancers.

### Adding genetic mutations to epigenetic methylation aberrations compromises their performance as clinical biomarkers

**Fig. 3:**
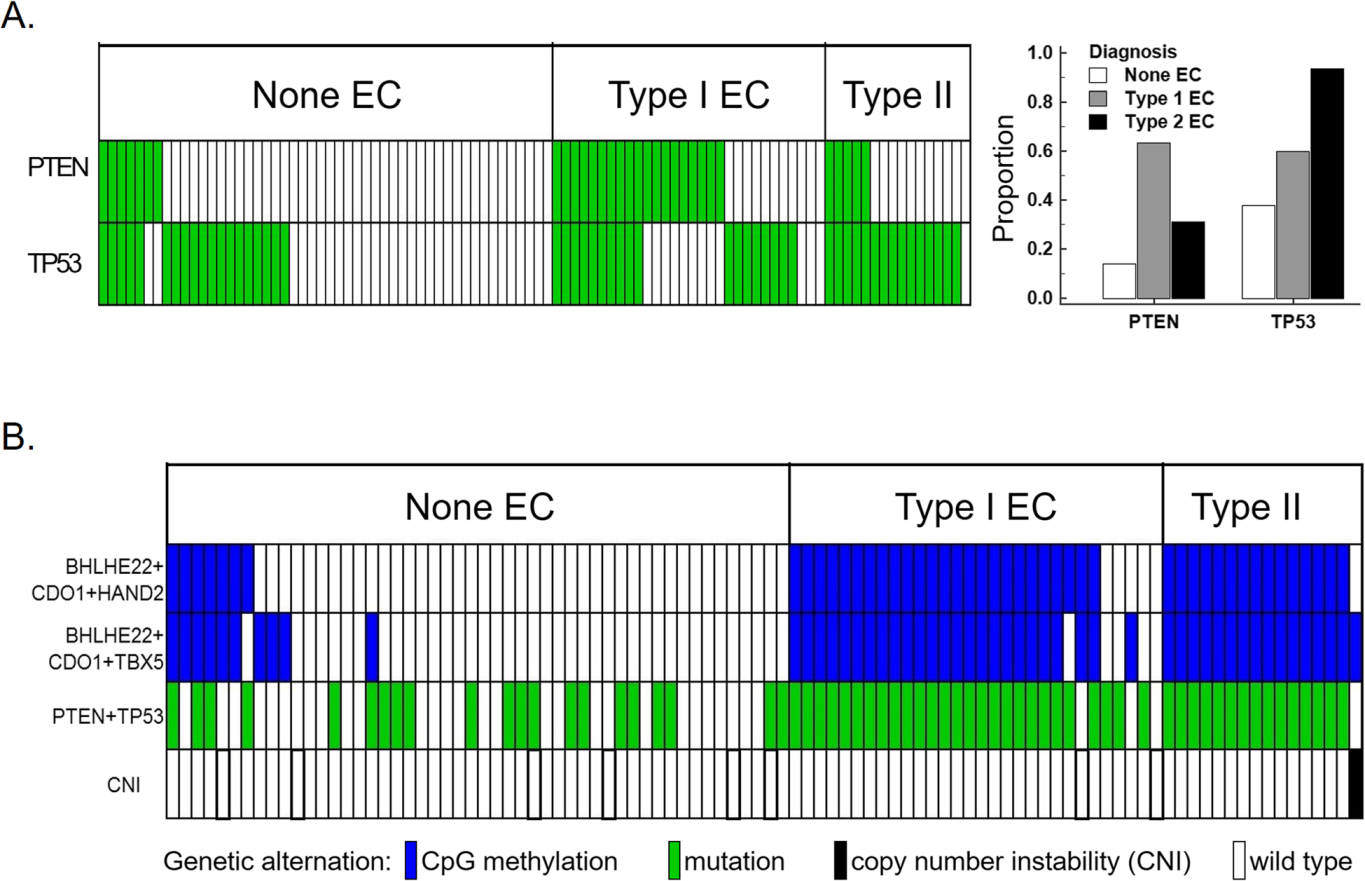
Comparison of genetic mutations (*PTEN* and *TP53*) and aberrantly DNA-methylated genes (*BHLHE22*, *CDO1*, *HAND2*, and *TBX5*) in cervical scrapings. **a** The distribution and frequency of *PTEN* and *TP53* gene mutations in non-endometrial cancer (non-EC), type I EC, and type II EC are shown. *PTEN* mutations were more frequently observed in type I EC. **b** Combination of DNA methylation and any genetic mutation in EC and non-EC. The absence of *PTEN* and *TP53* mutations and no DNA methylation of any of the four candidate genes were seen in one of the 16 type II EC samples. This sample also revealed unique copy number instability (CNI)

For clinical applications, any disease-correlated genetic alterations were called positive, as shown in Fig. 3. For example, the sensitivities and specificities of *PTEN* and *TP53* mutations for overall ECs were 52.2% and 86%, and 71.1% and 62%, respectively (Fig. 3b and Table 2). In addition to those genomic assays, we assessed whether three combinations of DNA methylation levels of the *BHLHE22*, *CDO1*, *TBX5*, and *HAND2* genes identified in our previous study [18] might provide the best performance for EC detection in terms of sensitivity and specificity. The sensitivities and specificities of combined DNA methylation were 87.0–89.1% (*BHLHE22*/*CDO1*/*HAND2*) and 80.0–86.0% (*BHLHE22*/*CDO1*/*TBX5*), respectively. Adding genetic mutations increased the detection sensitivity of type I, but not type II ECs, but the specificity was severely compromised because of the numerous mutations detected in patients without ECs (Fig. 3b).

**Table 2:** Performance of genetic mutations, and methylated gene combinations, in cervical scrapings

Variables	Non-endometrial cancer	Endometrial cancer	*P* value^a^
Number of cases	50	46	
Genetic mutation			
*PTEN*			< 0.001
Mutation	7 (14.0%)	24 (52.2%)	
Wild type	43 (86.0%)	22 (47.8%)	
*TP53*			< 0.001
Mutation	19 (38.0%)	33 (71.7%)	
Wild type	31 (62.0%)	13 (28.3%)	
Either mutation			< 0.001
Mutation	29 (58.0%)	42 (91.3%)	
Wild type	21 (42.0%)	4 (8.7%)	
DNA methylation			
*BHLHE22*+*CDO1* (cutoff value > − 0.2)^b^			< 0.001
High	6 (12.0%)	39 (84.8%)	
Low	44 (88.0%)	7 (15.2%)	
*BHLHE22*+*CDO1*+*HAND2* (cutoff value > 0.22)^b^			< 0.001
High	7 (14.0%)	40 (87.0%)	
Low	43 (86.0%)	6 (13.0%)	
*BHLHE22*+*CDO1*+*TBX5* (cutoff value > − 0.04)^b^			< 0.001
High	10 (20.0%)	41 (89.1%)	
Low	40 (80.0%)	5 (10.9%)	

^a^*P* values were calculated by the chi-square test

^b^Cutoff values were calculated by the maximum of Youden’s distance of receiver operating characteristic curve

## Discussion

Endometrial cancer (EC) is the most commonly diagnosed gynecologic malignancy, and incidences and mortality rates are increasing at an alarming pace [1]. EC already has a high prevalence, and its morbidity and mortality rates continue to increase; however, to our knowledge, no existing screening method can effectively detect either precancerous lesions or early-stage cancer. The need for improved screening is particularly high because when detected early, EC survival rates dramatically improve. EC is a heterogeneous disease [25], and the risks of EC development are highly influenced by genetic and non-hereditary factors [26]. In light of the increase of high-risk populations due to the current aging and obesity epidemics, women with risk factors such as postmenopausal bleeding, breast cancer treatment with hormone therapy, and a family history of Lynch syndrome desperately need novel and noninvasive molecular biomarkers that can detect EC early. In addition to being useful for genetic mutation testing [11, 27], cervical scrapings could also play a functional role in methylation-based screening [28] to facilitate the detection of EC. To that end, this study was the first to investigate whether combined testing of genetic mutations and epigenetic DNA methylation alterations from cervical scrapings would increase sensitivity and specificity for the detection of EC.

Our study provides supporting evidence that epigenetic biomarkers (i.e., a *BHLHE22*/*CDO1*-based panel) may be more useful than genetic mutation-based biomarkers for detecting EC. In the 96 cervical scrapings, we found high AUC-ROCs for four hypermethylated genes (*BHLHE22*, *CDO1*, *HAND2*, and *TBX5*, which were previously identified by our group [18]) in patients with type I and type II ECs compared with patients with normal endometrium and endometrial hyperplasia. These genes have low methylation levels in cervical lesions (Additional file 1: Table S1). We also demonstrated that three-gene panels of hypermethylated *BHLHE22*/*CDO1*/*HAND2* and *BHLHE22*/*CDO1*/*TBX5* were significantly different between patients with and without EC. By contrast, *PTEN* and *TP53* mutations were commonly found in cervical scrapings of healthy controls and those with benign gynecologic diseases. Clonal proliferations of nonmalignant cells and benign diseases have been described in the bone marrow and noncancerous tissue, and in endometriosis [29–32]. Although it these mutations might reflect benign or noncancerous endometrial lesions [10, 32], the mechanism underlying the mutational changes in normal endometrium and benign uterine lesions remains to be elucidated.

We also provide exploratory insights that ECs may share common epigenetic events, and that DNA methylation changes regardless of genetic heterogeneity and clinicopathology. ECs are conventionally classified into types I and II according to their clinicopathological characteristics and heterogeneity at the genetic level. In addition to these genetic events, the role of epigenetics combined with mutation-driven classification remains unknown. An important issue is whether different panels of DNA methylation profiles might be able to distinguish the two EC histotypes. Our previous study [18] identified *BHLHE22*, *CDO1*, and *CELF4* methylation in cervical scrapings as excellent molecular biomarkers for EC detection. When detecting type II ECs, 14 samples, except for one of the serous type (92.9%), showed hypermethylation of this three-gene panel. Our present study further confirmed that both type I and II ECs can be detected by a *BHLHE22*/*CDO1*-based methylation profile. *BHLHE22* (also known as *BHLHB5*) encodes a basic helix–loop–helix transcription factor and has been implicated in neural development, including cell growth, cell differentiation, and cell migration [33]. *CDO1* is a tumor suppressor gene, and methylation of its promoter region has been found in numerous cancers [34, 35]. Additionally, T-box transcription factor 5 (*TBX5*) is a member of a phylogenetically conserved family of genes involved in the regulation of developmental processes. The function of *TBX5* in cancer development is largely unclear [36–38]. *HAND2* is a progesterone-regulated stem cell polycomb group target gene that encodes a transcription factor expressed in the endometrial stroma to suppress estrogen-mediated signals. One previous study [14] reported that DNA methylation of *HAND2* could be a key step in EC development and, thus, could potentially be used as a biomarker for the early detection of ECs.

Today, mutations in *PTEN* and *TP53* are best documented in genetic lesions occurring in sporadic ECs. In our present and previous studies, *PTEN* mutations were detected in 63.3% of type I ECs and 31.2% of type II ECs [5, 39]. Up to 93.6% of type II and 63.3% of type I EC patients harbored detectable *TP53* mutations in their cervical scraping samples. Therefore, *TP53* mutations are not restricted to type II endometrial serous carcinomas; they are also present in a subset of type I endometrial endometrioid carcinomas. The potential relationship between mutations in *TP53* and *PTEN*, as observed in endometrial carcinoma, is still poorly known [40–42]. Furthermore, to ensure that genetic screening profiles are more informative, we assert that the mutation-based detection thresholds should be stricter, and that the criteria to determine cutoff points should be more specific. *PTEN* mutations seem to be of little importance in uterine cervical lesions [43]. Our supplementary data showed that *PTEN* and *TP53* mutations had been found in 13% and 9% of cervical squamous cell carcinoma cases, respectively (Additional file 1: Figure S2). Recently, Salk et al. reported that subclonal mutations in cancer evolutionary processes were ubiquitous and part of normal human aging. Therefore, great care must be taken to distinguish tumor-derived from age-associated mutations in high-sensitivity clinical cancer diagnostics [44].

Despite its notable findings, our study had several limitations. First, it was retrospective rather than prospective. Moreover, the samples examined were derived from patients with abnormal uterine bleeding. In a screening setting, endometrial premalignant lesions and cancers will include earlier stage cancers and asymptomatic women. Early detection and treatment prediction require further validation, both in prospective and unbiased cohorts. Additionally, we need to explore the value of combined genetic and epigenetic analyses of DNA obtained from cervical scrapings during a routine Pap test in asymptomatic women to detect hidden ECs. Second, cutoff values for candidate biomarker genes for research purposes might not be directly applicable to clinical settings or the wider population, thereby warranting further validation in larger, population-based studies. Third, we found mutation and methylation changes in cervical scrapings from women with normal endometrium and benign uterine diseases. These “background” readings could interfere with the sensitivity and specificity rates of the detection of ECs, necessitating some type of filtering. Finally, the highest methylation levels we detected were in fully cancerous tissues, atypical endometrial hyperplasia, or stage I diseases. Thus, future studies should concentrate on identifying biomarkers specific for these types of cells for early detection.

## Conclusions

In conclusion, we demonstrated promising epigenetic biomarkers in cervical scrapings for EC screening to triage women with abnormal uterine bleeding for invasive procedures. These epigenetic biomarkers could broaden the scope of Pap testing and potentially be employed to detect ECs in the early stage, when the disease is easiest to treat. The value of adding genetic- to epigenetic-based biomarkers to detect EC requires further investigation.

## Supplementary information

**Additional file 1: Table S1.** Performance of methylated gene combinations to detect endometrial cancer in scrapings of cervical lesions. **Figure S1.** The analytical sensitivity of target genes and limit of *COL2A1* detection. Linear regression analysis was performed to assess assay linearity. **(A and B)** The ΔCp values and serial percentages of methylated DNA showed a high correlation (R^2^ = 0.998 and 0.99 for *BHLHEE22* and *CDO1*, respectively). The mean ΔCp values of 0.1% methylated DNA were 7.87 and 7.45 for *BHLHEE22* and *CDO1*, respectively. Additionally, when the DNA temple was 118 copies, the mean Cp value of *COL2A1* was 35.8 **(C)**. Therefore, we defined clinical samples with Cp values of *COL2A1* > 36 as not detectable. **Figure S2.** *PTEN* and *TP53* mutations in cervical squamous cell carcinoma. We visualized these two genetic mutations from cBioPortal (https://www.cbioportal.org/). Copy number alteration data including 278 samples from The Cancer Genome Atlas and the PanCancer Atlas studies were chosen for mutation analysis

## Abbreviations

AUC: Area under a receiver-operating characteristic curve
CI: Confidence interval
CNA: Copy number abnormality
CNI: Copy number instability
Cp: Crossing point
EC: Endometrial cancer
EH: Endometrial hyperplasia
*HAND2*: Heart and neural crest derivatives expressed 2
Pap: Papanicolaou
*PTEN*: Phosphatase and tensin homologue
qMS-PCR: Quantitative methylation-specific polymerase chain reaction
ROC: Receiver operating characteristic
TCGA: The Cancer Genome Atlas
