# Supplementary material for: Combined genetic mutations and DNA-methylated genes as biomarkers for endometrial cancer detection from cervical scrapings
Source: Clin Epigenetics. 2019 Nov 28;11:170. doi: 10.1186/s13148-019-0765-3 (PMC6883641; doi:10.1186/s13148-019-0765-3)

Additional files

Table S1. Performance of methylated gene combinations in scrapings of cervical lesions.

**Variables**	**CIN 2/CIN 3**	**CIS/SCC**
Number of cases	10	9
DNA Methylation		
BHLHE22+CDO1		
High	0	1 (11.1%)
Low	10 (100%)	8 (88.9%)
BHLHE22+CDO1+HAND2		
High	0	0
Low	10 (100%)	9 (100%)
BHLHE22+CDO1+TBX5		
High	0	1 (11.1%)
Low	10 (100%)	8 (88.9%)

The cut-off values were listed in Table 2.

CIN, Cervical intraepithelial neoplasia; CIS, cervical carcinoma in situ; SCC, Squamous Cell Carcinoma.

Figure S1. The analytical sensitivity of target genes and limit of *COL2A1* detection. Linear regression analysis was performed to assess assay linearity. **(A and B)** The ΔCp values and serial percentages of methylated DNA showed a high correlation (R^2 = 0.998 and 0.99 for *BHLHEE22* and *CDO1*, respectively). The mean ΔCp values of 0.1% methylated DNA were 7.87 and 7.45 for *BHLHEE22* and *CDO1*, respectively. Additionally, when the DNA temple was 118 copies, the mean Cp value of *COL2A1* was 35.8 **(C)**. Therefore, we defined clinical samples with Cp values of *COL2A1* >36 as not detectable.

A.

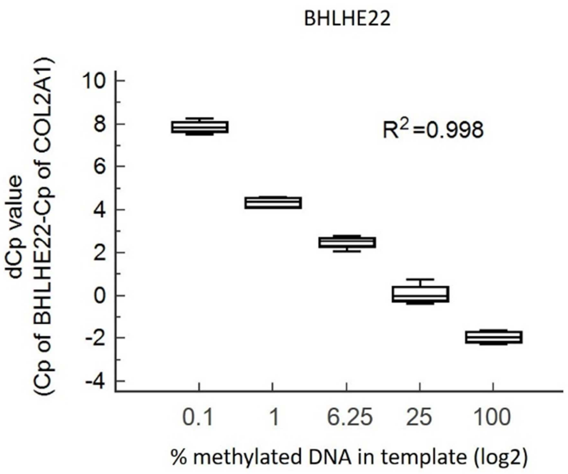

B.

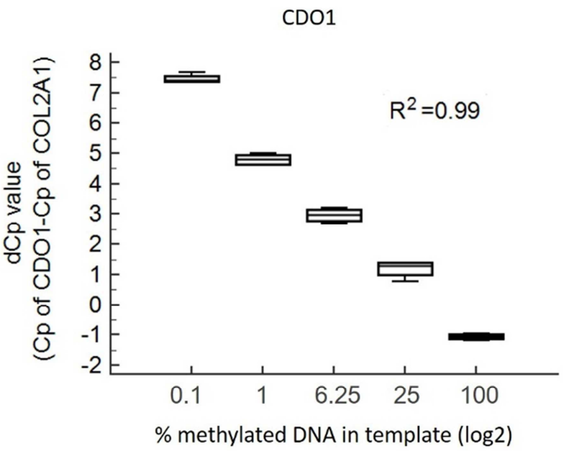

C.

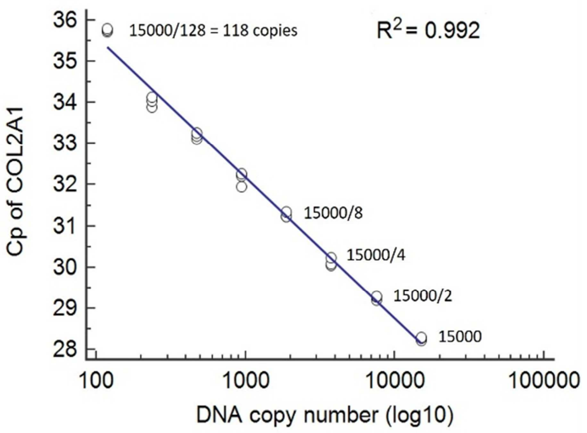

Figure S2. *PTEN* and *TP53* mutations in cervical squamous cell carcinoma. We visualized these two genetic mutations from cBioPortal (https://www.cbioportal.org/). Copy number alteration data including 278 samples from The Cancer Genome Atlas and the PanCancer Atlas studies were chosen for mutation analysis.

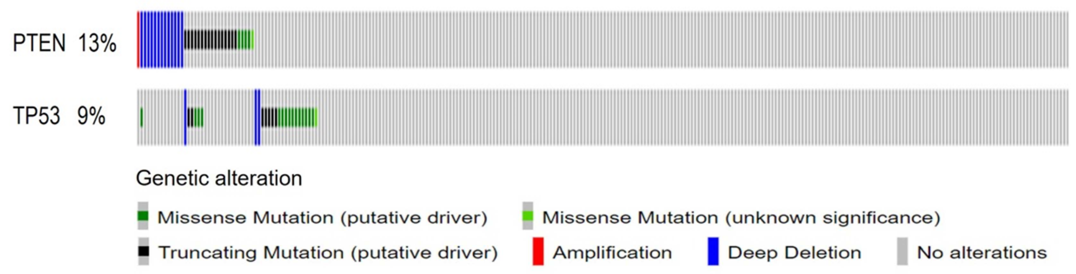

Supplement: Supplementary file 1 — Additional file 1: Table S1. Performance of methylated gene combinations to detect endometrial cancer in scrapings of cervical lesions. Figure S1. The analytical sensitivity of target genes and limit of COL2A1 detection. Linear regression analysis was performed to assess assay linearity. (A and B) The ΔCp values and serial percentages of methylated DNA showed a high correlation (R2 = 0.998 and 0.99 for BHLHEE22 and CDO1, respectively). The mean ΔCp values of 0.1% methylated DNA were 7.87 and 7.45 for BHLHEE22 and CDO1, respectively. Additionally, when the DNA temple was 118 copies, the mean Cp value of COL2A1 was 35.8 (C). Therefore, we defined clinical samples with Cp values of COL2A1 > 36 as not detectable. Figure S2. PTEN and TP53 mutations in cervical squamous cell carcinoma. We visualized these two genetic mutations from cBioPortal (https://www.cbioportal.org/). Copy number alteration data including 278 samples from The Cancer Genome Atlas and the PanCancer Atlas studies were chosen for mutation analysis [file 13148_2019_765_MOESM1_ESM.pdf]
